# In-depth characterization of breast cancer tumor-promoting cell transcriptome by RNA sequencing and microarrays

**DOI:** 10.18632/oncotarget.5810

**Published:** 2015-11-03

**Authors:** Maurizio Callari, Alessandro Guffanti, Giulia Soldà, Giuseppe Merlino, Emanuela Fina, Elena Brini, Anna Moles, Vera Cappelletti, Maria Grazia Daidone

**Affiliations:** ^1^ Department of Experimental Oncology and Molecular Medicine, Fondazione IRCCS Istituto Nazionale dei Tumori, Milan, Italy; ^2^ Genomnia Srl, Lainate, Milan, Italy; ^3^ Department of Biomedical Sciences, Humanitas University, Rozzano, Milan, Italy; ^4^ Humanitas Clinical and Research Center, Rozzano, Milan, Italy

**Keywords:** tumor-promoting cells, breast cancer, gene expression, ncRNAs, cancer stem cells

## Abstract

Numerous studies have reported the existence of tumor-promoting cells (TPC) with self-renewal potential and a relevant role in drug resistance. However, pathways and modifications involved in the maintenance of such tumor subpopulations are still only partially understood. Sequencing-based approaches offer the opportunity for a detailed study of TPC including their transcriptome modulation. Using microarrays and RNA sequencing approaches, we compared the transcriptional profiles of parental MCF7 breast cancer cells with MCF7-derived TPC (i.e. MCFS). Data were explored using different bioinformatic approaches, and major findings were experimentally validated. The different analytical pipelines (Lifescope and Cufflinks based) yielded similar although not identical results. RNA sequencing data partially overlapped microarray results and displayed a higher dynamic range, although overall the two approaches concordantly predicted pathway modifications. Several biological functions were altered in TPC, ranging from production of inflammatory cytokines (i.e., IL-8 and MCP-1) to proliferation and response to steroid hormones. More than 300 non-coding RNAs were defined as differentially expressed, and 2,471 potential splicing events were identified. A consensus signature of genes up-regulated in TPC was derived and was found to be significantly associated with insensitivity to fulvestrant in a public breast cancer patient dataset. Overall, we obtained a detailed portrait of the transcriptome of a breast cancer TPC line, highlighted the role of non-coding RNAs and differential splicing, and identified a gene signature with a potential as a context-specific biomarker in patients receiving endocrine treatment.

## INTRODUCTION

There is substantial evidence to support the presence of a subpopulation of tumor-promoting cells (TPC) in both hematologic and solid tumors with self-renewal and asymmetric division capabilities. The proposed model looks at TPC as responsible for treatment failure due to their resistance to anticancer drugs and due to the inability of the presently employed drugs to specifically target the TPC subpopulation [1–4].

Identification and enumeration of such cells is difficult, their phenotypes are poorly defined and no specific biomarker allows a clear distinction of TPC, although several markers have been proposed [5]. The assay largely recognized as the gold standard to define a population of cells with tumor-initiating ability consists of xenotransplantation of serially diluted number of cells in immunocompromised mice (i.e., NOD/SCID or NOD-scid IL2Rgnull mice) [6], and an optimal tool for isolating breast TPC from clinical tumors is an *in vitro* functional approach (i.e., sphere formation) [7].

In breast and other tumor types, much effort has been made to identify the pathways involved in maintenance of the TPC phenotype and to tackle possible TPC-specific targets with therapeutic potential. Among others, Notch [8, 9] and Hedgehog pathways [10] have been suggested as central pathways for TPC maintenance. More recently, a role for NF-κB NF-kappaB-related genes [11, 12] and for inflammatory cytokines [13, 14] has been proposed, also linking stemness with epithelial-mesenchymal transition [15, 16].

Accumulating evidence in other malignancies suggests that also poorly characterized non-coding RNAs (ncRNAs) could have a role in cancer [17] and in the maintenance of a stem-like phenotype [18]. In addition, the isoform composition of the coding transcript population has been demonstrated to be important in stem cell biology [19, 20] and cancer [21]. Massive RNA sequencing (RNA-seq) allows an in-depth transcriptome analysis, which includes the annotation and evaluation of differential expression for both the coding and non-coding transcripts and the identification and quantitative evaluation of alternative splicing events. This type of analysis proved to extend biological knowledge and to identify additional biomarkers [22].

We previously reported the isolation and *in vitro* propagation of highly tumorigenic mammospheres isolated from the MCF7 breast cancer cell line (commonly defined as MCFS) [23]. In the present study, we obtained gene expression profiles of MCFS and parental MCF7 cell lines using Illumina microarrays and SOLiD RNA-seq. Different analytical approaches for RNA-seq were used and the results compared. Differentially expressed coding and non-coding RNAs, deregulated pathways and alternative splicing events were identified by specific bioinformatic approaches and validated *in vitro*. Finally, the significance of the TPC gene signature derived from our model was confirmed in a cohort of endocrine therapy-treated breast cancer patients.

## RESULTS AND DISCUSSION

### Comparison of RNA-seq and microarray signals

Transcriptome analysis of tumor-promoting mammospheres (MCFS) and of the parental breast cancer cell line MCF7 was run in triplicate on microarrays and as a single experiment using RNA-seq after linear isothermal DNA amplification. In microarray data, 9,283 probes, corresponding to the same number of genes, were retained after normalization and filtering.

**Figure 1:**
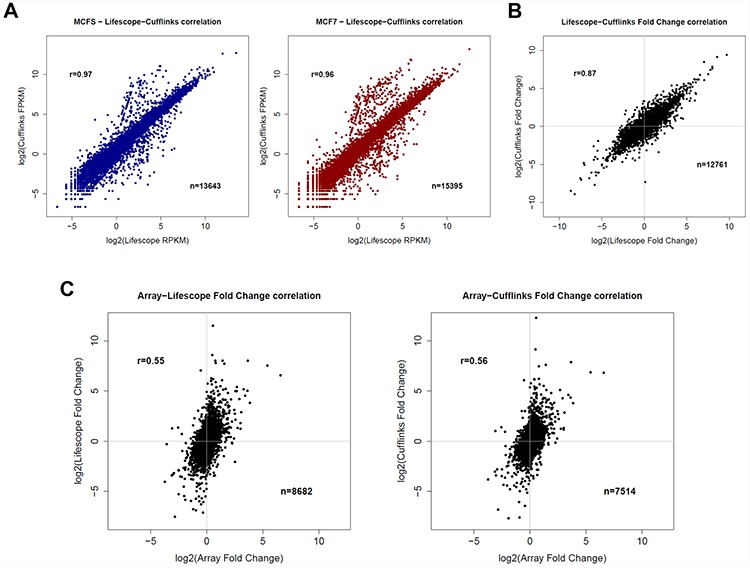
Comparison of different transcriptomic data **A.** Correlation of RNA-seq gene quantification from MCFS (left) or MCF7 (right) cells obtained with the two pipelines used (Lifescope or Cufflinks). **B.** Correlation of log fold-changes between MCFS and MCF7 cells obtained with the two pipelines used (Lifescope or Cufflinks). **C.** Correlation of log fold-changes obtained using Arrays or RNA-seq transcriptomic data, processed with the Lifescope pipeline (left) or Cufflinks pipeline (right).

Since using only one method for the analysis of RNA-seq datasets can result in a suboptimal analysis, especially when working with a cancer transcriptome, we decided to apply two different methods for the absolute quantification of gene expression after the genome mapping step, i.e., Lifetech Lifescope 2.5.1 pipeline and the TopHat/Cufflinks method (version 1.0.2), using as a common reference gene annotation the UCSC RefSeq dataset. Supplementary Table S1 summarizes the number of genes with non-zero quantification for expression values processed according to the two pipelines. Ninety-four percent of the genes detected by Cufflinks was also identified as expressed by Lifescope, whereas the latter globally identified a higher number of genes. For each library, gene expression levels, measured as RPKM or FPKM (Reads/Fragments Per Kilobase of exon per Million fragments mapped) for each of the two experiments, were correlated, displaying good correlation coefficients (R = 0.97/0.96) (Figure 1A).

We next correlated fold changes (FC) between MCFS and MCF7 cells obtained with the two analytical pipelines. As for RPKM and FPKM values, a good correlation was found, but the correlation coefficient (R = 0.89) was affected by large expression differences present between the two cell lines (up to 1000-fold changes). Usually, fold changes higher than 2 are already considered to be biologically relevant, but, as can be seen in Figure 1B, such an extent of modulations seems to be less reproducible when using two different processing procedures, even though starting from the same raw sequence data.

Fold changes obtained from RNA-seq were then correlated with the results elaborated from microarray data for common genes. As expected, lower correlations were obtained with RNA-seq data, independently of the pipeline used, clearly showing a higher dynamic range, in fold change terms, than microarrays (Figure 1C). A similar platform comparison, run on CD44+/CD24− cancer stem isolated from primary ER-positive breast cancer cells [24] reported a good match between next generation transcriptome sequencing and microarrays, but no details were given on the analytical pipeline used for RNA-seq data.

### Identification of enriched gene sets and functional validation of data

Microarray as well as RNA-seq expression data were subjected to a Gene Set Enrichment Analysis (GSEA) in order to provide a robust way to compare elaborated gene expression data sets obtained with different platforms and to highlight biologically meaningful pathways modulated in MCFS compared to MCF7 cells. For RNA-seq data, both data analysis procedures were considered in order to investigate their reliability by comparison with array data. For all data, genes were ordered according to fold changes and array data were ordered according to GSEA statistics. Data analyzed with Cufflinks, besides fold changes, were ordered according to the ranking suggested by the Cuffdiff statistics. Enrichment for functionally related genes was tested across a collection of 4,850 curated gene sets (C2 collection), and a summary of obtained results is reported in Supplementary Table S2. Similar results were obtained for array data ranked using either fold change or GSEA statistics. Gene quantification using Cufflinks and ranking using Cuffdiff statistics slightly outperformed the other methods in terms of number of significantly enriched gene sets and concordance with array data. Consequently, we focused on GSEA statistics ranked array data and Cuffdiff ranked RNA-seq data to elaborate a biological interpretation of the coding genes modulated in MCFS TPC.

To obtain a meaningful interpretation of the findings, our approach was to distribute the gene sets in different categories, linking them to a possible biological function. Such biofunctions, which were differentially modulated in MCFS compared with MCF7 cells are listed in Table 1, together with information of the single gene sets supporting them. The main biofunctions are reported below, together with results of validation experiments.

**Table 1:** Enriched biological functions

Biofunction	Description	n° Gene sets associated	Gene Sets	Enriched in:	FDR Array	FDR Cufflinks
*Cell Cycle, Proliferation*	Gene sets involved in cells cycle and proliferation	7	KONG_E2F3_TARGETS	MCF7	< 1e-4	< 1e-4
			ISHIDA_E2F_TARGETS	MCF7	< 1e-4	< 1e-3
			ZHOU_CELL_CYCLE_GENES_IN_IR_RESPONSE_6HR	MCF7	< 1e-4	< 1e-2
			ROSTY_CERVICAL_CANCER_PROLIFERATION_CLUSTER	MCF7	< 1e-4	< 1e-2
			MOLENAAR_TARGETS_OF_CCND1_AND_CDK4_DN	MCF7	< 1e-4	< 1e-2
			ZHOU_CELL_CYCLE_GENES_IN_IR_RESPONSE_24HR	MCF7	< 1e-4	< 1e-3
			ZHANG_TLX_TARGETS_ 60HR_DN	MCF7	< 1e-3	< 1e-4
*Endocrine Therapy Sensitivity*	Gene sets involved in response to various endocrine therapy regimens in which many tumor cells manifest resistance, either de novo or acquired during the treatment	8	CREIGHTON_ENDOCRINE_THERAPY_ RESISTANCE_5	MCFS	not tested	< 1e-2
			DOANE_BREAST_CANCER_CLASSES_UP	MCFS	> 0.01	< 1e-2
			FARMER_BREAST_ CANCER_APOCRINE_VS_LUMINAL	MCFS	> 0.01	< 1e-2
			FARMER_BREAST_ CANCER_APOCRINE_ VS_BASAL	MCFS	> 0.01	< 1e-2
			BECKER_TAMOXIFEN_RESISTANCE_UP	MCFS	> 0.01	< 1e-4
			FRASOR_RESPONSE_ TO_ESTRADIOL_UP	MCF7	< 1e-2	< 1e-2
			DUTERTRE_ESTRADIOL_RESPONSE_6HR_DN	MCFS	< 1e-2	> 0.01
			DOANE_BREAST_CANCER_ ESR1_DN	MCFS	< 1e-4	not tested
*Immune Response*	Gene sets involved in several mechanism of immune response	22	KEGG_CYTOKINE_CYTOKINE_RECEPTOR_INTERACTION	MCFS	< 1e-4	< 1e-2
			SANA_TNF_SIGNALING_UP	MCFS	< 1e-2	< 1e-3
			ZHENG_IL22_SIGNALING_UP	MCFS	< 1e-3	not tested
			WINZEN_DEGRADED_ VIA_KHSRP	MCFS	< 1e-2	not tested
			KEGG_COMPLEMENT_AND_COAGULATION_CASCADES	MCFS	< 1e-2	not tested
			BENNETT_SYSTEMIC_LUPUS_ERYTHEMATOSUS	MCFS	< 1e-2	not tested
			KIM_GLIS2_TARGETS_UP	MCFS	< 1e-2	not tested
			ZHANG_RESPONSE_TO_IKK_INHIBITOR_AND_TNF_UP	MCFS	< 1e-4	> 0.01
			HINATA_NFKB_TARGETS_KERATINOCYTE_UP	MCFS	< 1e-2	> 0.01
			ICHIBA_GRAFT_VERSUS_HOST_DISEASE_35D_UP	MCFS	< 1e-2	> 0.01
			LINDSTEDT_DENDRITIC_CELL_MATURATION_A	MCFS	< 1e-2	> 0.01
			HINATA_NFKB_TARGETS_FIBROBLAST_UP	MCFS	< 1e-2	> 0.01
			LI_INDUCED_T_TO_NATURAL_KILLER_UP	MCFS	< 1e-2	> 0.01
			EINAV_INTERFERON_ SIGNATURE_IN_CANCER	MCFS	> 0.01	< 1e-4
			SANA_RESPONSE_TO_ IFNG_UP	MCFS	> 0.01	< 1e-4
			BROWNE_INTERFERON_RESPONSIVE_GENES	MCFS	> 0.01	< 1e-4
			DER_IFN_ALPHA_ RESPONSE_UP	MCFS	> 0.01	< 1e-2
			GAVIN_FOXP3_TARGETS_CLUSTER_P4	MCFS	> 0.01	< 1e-2
			BOSCO_INTERFERON_ INDUCED_ANTIVIRAL_ MODULE	MCFS	> 0.01	< 1e-2
			DAUER_STAT3_TARGETS_DN	MCFS	> 0.01	< 1e-4
			CROONQUIST_IL6_ DEPRIVATION_DN	MCF7	< 1e-4	< 1e-3
			CROONQUIST_NRAS_ SIGNALING_DN	MCF7	< 1e-4	< 1e-2
*Epigenetic Control*	Gene sets involved in altered methylation, acetylation status as a possible epigenetic mechanism of selection during tumorigenesis	9	MARTENS_TRETINOIN_RESPONSE_UP	MCFS	< 1e-2	< 1e-4
			MISSIAGLIA_REGULATED_BY_METHYLATION_UP	MCFS	< 1e-4	< 1e-4
			LIANG_SILENCED_BY_METHYLATION_2	MCFS	< 1e-4	not tested
			KIM_LRRC3B_TARGETS	MCFS	> 0.01	< 1e-4
			SATO_SILENCED_BY_METHYLATION_IN_PANCREATIC_CANCER_1	MCFS	> 0.01	< 1e-2
			HELLER_HDAC_TARGETS_SILENCED_BY_METHYLATION_DN	MCFS	> 0.01	< 1e-2
			MIKKELSEN_NPC_HCP_WITH_H3K4ME3_AND_H3K27ME3	MCFS	> 0.01	< 1e-2
			MISSIAGLIA_REGULATED_BY_METHYLATION_DN	MCF7	< 1e-4	< 1e-2
			ZHONG_RESPONSE_TO_AZACITIDINE_AND_TSA_DN	MCF7	< 1e-2	> 0.01
*Cholesterol Metabolism*	Gene sets involved in cholesterol metabolism pathways	6	REACTOME_CHOLESTEROL_BIOSYNTHESIS	MCFS	< 1e-3	not tested
			UZONYI_RESPONSE_TO_LEUKOTRIENE_AND_THROMBIN	MCFS	< 1e-3	not tested
			LIAN_LIPA_TARGETS_3M	MCFS	< 1e-3	not tested
			GARGALOVIC_RESPONSE_TO_OXIDIZED_PHOSPHOLIPIDS_GREEN_UP	MCFS	< 1e-2	not tested
			SCHMIDT_POR_TARGETS_IN_LIMB_BUD_UP	MCFS	< 1e-4	> 0.01
			GARGALOVIC_RESPONSE_TO_OXIDIZED_PHOSPHOLIPIDS_BLACK_UP	MCFS	< 1e-2	> 0.01
*Undifferentiation*	Gene sets associated with cells undifferentiation status	7	WIERENGA_STAT5A_TARGETS_GROUP2	MCFS	< 1e-2	not tested
			LIM_MAMMARY_LUMINAL_PROGENITOR_UP	MCFS	< 1e-2	> 0.01
			BURTON_ADIPOGENESIS_PEAK_AT_0HR	MCFS	> 0.01	< 1e-2
			PLASARI_TGFB1_TARGETS_10HR_UP	MCFS	< 1e-2	> 0.01
			BURTON_ADIPOGENESIS_10	MCFS	< 1e-2	> 0.01
			LENAOUR_DENDRITIC_CELL_MATURATION_DN	MCFS	< 1e-2	> 0.01
			MAHADEVAN_RESPONSE_TO_MP470_DN	MCF7	< 1e-2	not tested
*Growth factor response*	Gene sets involved in respose to different growth factors	9	SMID_BREAST_CANCER_ ERBB2_UP	MCFS	< 1e-4	< 1e-4
			NAGASHIMA_NRG1_ SIGNALING_UP	MCFS	< 1e-3	not tested
			NAGASHIMA_EGF_SIGNALING_UP	MCFS	< 1e-2	not tested
			PEDERSEN_METASTASIS_BY_ERBB2_ISOFORM_1	MCFS	< 1e-4	> 0.01
			PACHER_TARGETS_OF_IGF1_AND_IGF2_UP	MCFS	< 1e-4	< 1e-4
			AMIT_EGF_RESPONSE_60_MCF10A	MCFS	< 1e-2	not tested
			ZWANG_CLASS_3_TRANSIENTLY_INDUCED_BY_EGF	MCFS	< 1e-2	not tested
			XU_GH1_AUTOCRINE_ TARGETS_DN	MCF7	< 1e-2	< 1e-2
			KOBAYASHI_EGFR_SIGNALING_24HR_DN	MCF7	< 1e-2	> 0.01

#### Proliferation

As expected, based on the growth kinetics of the two cell types, genes associated to cell cycle progression, DNA replication and proliferation were expressed at lower levels in MCFS cells. Whereas the low proliferative potential of TPC is well known, no enrichments in proliferation related –genes has been reported in other studies comparing gene expression profiles of CD44+/CD24– breast cancer stem cells with the remaining bulk tumor cells [24] suggesting that the experimental conditions chosen for MCFS enrichment may play a role.

#### Endocrine therapy sensitivity

**Figure 2:**
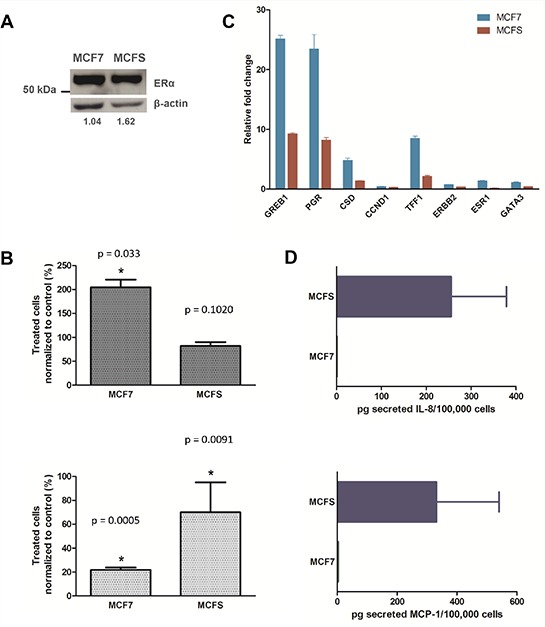
MCFS cell are less sensitive to E2 and fulvestrant stimulation and secrete higher quantities of IL-8 and MCP-1 compared to than MCF7 cells **A.** Western blotting analysis for ERα expression. ERα protein levels were normalized to a loading control. Numbers reported below gel images were obtained by densitometric analysis and represent the relative expression level of ERα normalized to β-actin expression level. **B.** Effect of 17β-estradiol (top) and fulvestrant (bottom) on cell growth. Cells were exposed to 10^−8^ M 17β-estradiol or fulvestrant for 6 days, and their growth was evaluated by direct cell counting. Cell growth of treated cells was expressed as percentage of that of untreated cells. Bar charts represent a mean ± CV% of 3 experimental replicates (*P < 0.05 by two-tailed Student's *t* test). **C.** Relative expression of ER-related genes in response to 17β-estradiol exposure. The relative expression levels of ER-related genes were measured after 48 h of exposure to 10^−8^ M 17β-estradiol by quantitative real-time PCR analysis and normalized to *RPL13A* expression level. Relative fold changes calculated by the ΔΔCt method refer to control cells. Bars represent mean ± SD relative fold changes, derived from 3 technical replicates. **D.** Quantification of secreted IL-8 (top) and MCP-1 (bottom) in conditioned media. The absolute quantity of cytokines secreted in culture media was measured by ELISA kit assays and normalized to the number of viable cells. Bars represent mean ± SD of cytokine levels (picograms per 10^5^ cells) derived from 3 technical replicates.

Gene sets up- and down-regulated after treatment of MCF7 cells with 17β-estradiol [25, 26] were found significantly enriched in the MCF7 and MCFS phenotype, respectively (Table 1). It is noteworthy that despite a distinct regulation of genes associated with estrogenic stimulation in the parental MCF7 cell line and in the derived TPC cells (Figure 2C), the estrogen receptor (ER) itself was expressed at comparable levels (Figure 2A). Such a gene expression pattern suggested the acquirement of an estrogen-insensitive phenotype in the MCFS, a hypothesis that was experimentally verified. Estrogen insensitivity has already been reported in the literature for CD44+/CD24– cells purified from human tumors, which were described as ER-negative also when deriving from ER-positive tumors [27]. Treatment with 10^−8^ M 17β-estradiol for 6 days caused almost a doubling in the MCF7 cell growth rate compared to untreated cells (*P* = 0.033), whereas as expected based on gene expression data, estradiol had no significant effect on MCFS cell growth (Figure 2B). Consistent with the loss of estrogen sensitivity in the MCFS cells, also treatment with the pure antiestrogen fulvestrant displayed a higher cytostatic effect in MCF7 cells than in MCFS (80% vs 30% growth inhibition, respectively). Such results suggest an insensitivity of MCFS cells to estrogenic stimulations and a limited response to treatment with antiestrogen, in agreement with impairment on estrogenic response in MCFS cells.

In order to provide a further confirmation of the impairment in ER-mediated response to estrogens in MCFS cells, we evaluated the expression levels of typically ER-related genes after exposure of the cells to estradiol. In agreement with the proliferative behavior of these cells in response to estrogens, also induction of the estrogen-regulated genes GREB1, PGR, CSD and TFF1 was stronger in MCF7 cells than in MCFS, with a more than two-fold difference depending on the considered gene (Figure 2C).

In accord with literature data demonstrating that TPCs are intrinsically resistant to conventional chemotherapeutic agents and to radiotherapy [4, 28, 29], we provided evidence that such cells are also less sensitive to competitive ER antagonists, such as selective estrogen receptor down regulators, suggesting that the outgrowth of a subpopulation of cells with tumor-promoting properties might be responsible for hormone therapy resistance in breast cancer. The presence of ERα, the main ligand-mediated transcriptional factor responsible for estrogenic effects in breast cancer, still guides the choice of endocrine treatments, although it is known to represent a better predictor for endocrine insensitivity (if negative) than an optimal sensitivity biomarker [30]. In fact, acquired, but also de novo resistance to endocrine therapy is often observed in tumors defined as ER+. Here we show new data suggesting that treatment with fulvestrant might fail due to the presence of cells with tumor-promoting characteristics such as our MCFS cells.

It is also worth to mention that paradoxically the TPC population (defined as enriched for expression of ESA^+^CD44^+^CD24^low^ cells), increases in response to estradiol treatment due to paracrine stimulation by non-TPC ER-positive cells mainly through EGFR [31].

#### Immune response

Genes associated with immune response were expressed at higher levels by MCFS cells. In keeping with previous reports even in different TPC models [11, 12], our result suggests a central role for NF-κB signaling in MCFS cells, as many pathways and genes regulated by this transcription factor were found up-regulated. Of particular note, CXCL8, whose expression is regulated by NF-κB and which is involved in self-renewal of mammospheres [32], showed higher expression in MCFS than in MCF7 cells. Therefore, using an ELISA assay we explored at the protein level release of the interleukin in the culture medium. We also validated *in vitro*, by the ELISA assay, the production of other cytokines (TNF and MCP-1), confirming the gene expression data (Figure 2D).

#### Other biological functions

Differentially enriched gene sets also suggested a role of epigenetic mechanisms, cholesterol metabolism and growth factor (mainly epidermal growth factor) response in the maintenance of “stemness”. Finally, several gene sets supported the undifferentiated state of MCFS cells (Table 1).

### Identification and validation of differentially expressed ncRNAs

To identify differentially expressed IncRNAs, we compared transcripts derived from Cufflinks analysis with ncRNAs represented in the RefSeq database (June 2013 release) and with those annotated in the ENCODE/GENCODE v.7 lncRNA catalog (June 2013) dataset. This way a total of 331 and 398 noncoding transcripts were respectively identified and were manually annotated in subclasses (Table 2 and Supplementary Table S3).

**Table 2:** Differentially expressed (DE) ncRNAs identified by RNA-Seq

ncRNAs	RefSeq ncRNAs^1^			GENCODE/ENCODE ncRNAs^2^		
	DE	UP in MCFS^3^	DOWN in MCFS^4^	DE	UP in MCFS^3^	DOWN in MCFS^4^
**Total DE transcripts**	331	86	245	398	197	201
**Processed transcripts**	198	43	155	6	3	3
**Expressed pseudogenes**	26	12	14	7	5	2
**lincRNAs**	50	16	34	215	102	113
**Antisense**	43	14	19	159	84	75
**Sense Intronic**	7	1	6	7	1	6
**Sense overlapping**	3	1	2	3	1	2
**Small RNAs**	9	0	9	1	1	0

lincRNAs: long intergenic noncoding RNAs

1 noncoding RNAs annotated in RefSeq, release June 2013

2 noncoding RNAs annotated in GENCODE/ENCODE v.17 June 2013

3 log2 fold change > 1.5, *p* < 0.01

4 log2 fold change < −1.5, *p* < 0.01

**Figure 3:**
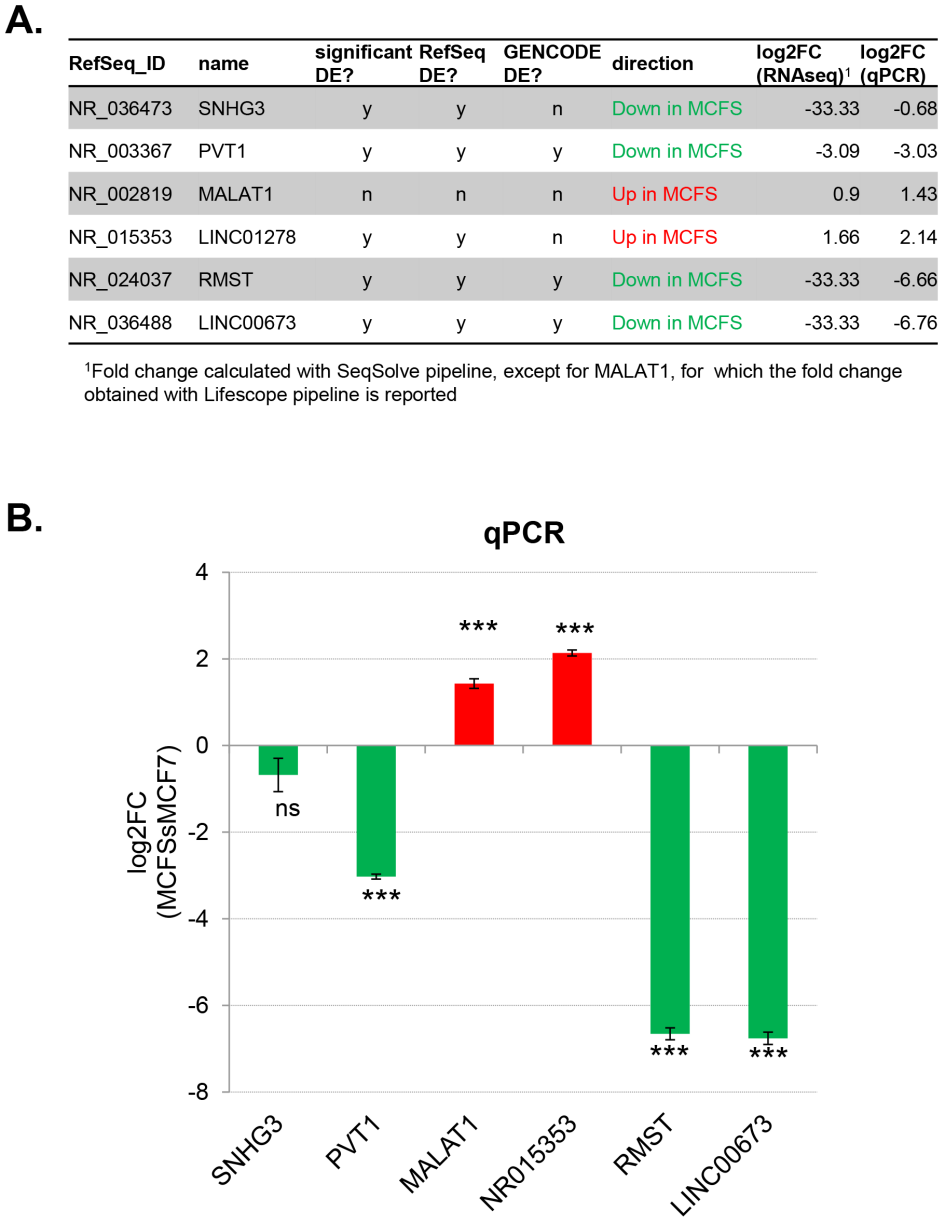
Selection and validation of differentially expressed non-coding RNAs **A.** Table listing the 6 ncRNAs selected for experimental validation. **B.** Differential expression of candidate ncRNAs by semi-quantitative real-time RT-PCR. Bars represent mean ± SD of 3 technical replicates. Fold change between MCFS and MCF7 cells were calculated with the ΔΔCt method, setting the MCF7 sample as 1. The glucuronidase (GUSB) housekeeping gene was used as internal control for normalization. The results were analyzed by unpaired *t*-test: ***P < 0.0001; ns, not significant.

Five ncRNAs were selected for experimental validation: four of them were down-regulated in MCFS compared to MCF7 cells (SNHG3, PVT1, RMST, and LINC00673), whereas the remaining one (LINC01278) was up-regulated. In addition, we chose to validate the differential expression of the long ncRNA MALAT1, previously reported to be highly expressed also in breast cancer tissue [33], as it showed a two-fold increased expression in MCFS compared to MCF7 cells by RNA-seq, but without reaching statistical significance (Figure 3A). Real-time RT-PCR assays substantially confirmed the RNA-seq results in all cases except SNHG3 (Figure 3B), which did not show a statistically significant differential expression between the two cell lines. This might be due, at least in part, to the design of the corresponding RT-PCR assay, which specifically amplified only one transcriptional isoform of SNHG3 (i.e., NR_036473). On the contrary, the differential expression of MALAT1 was more marked in the real-time assay (about a 2.7-fold increase in MCFS compared to MCF7 cells) than in RNA-seq data.

### Identification and validation of splicing events

Alternatively spliced genes were identified using a reciprocal junction analysis as implemented in the AltAnalyze software [34], starting from the exon level expression quantification and exon junction files generated by the Lifescope pipeline. A total of 2,471 reciprocal junctions showed a significant splicing score (ASPIRE > 0.2 or < 0.2). A filtering step was subsequently applied in order to identify the most robust and biologically meaningful splicing events (Supplementary Figure S1). A complete list of such filtered events is reported in Supplementary Table S4. Among the genes that were consistently detected as displaying significant differential alternative splicing events, we selected 3 for experimental validation: the myoferlin (MYOF) exon 18 alternative splicing, the inclusion/skipping of SRFS10 exon 3, and a fusion between the VMP1 and RPS6KB1 genes. In particular, AltAnalyze predictions evidenced a significant decrease of both MYOF exon 18 and SRSF10 exon 3 inclusion events in MCFS compared to MCF7 cells, as well as an increase in fusion events between multiple RPS6KB1 exons and VMP1 exon 12 (Supplementary Table S4).

To verify the predictions, a specific measurement of the relative abundance of transcripts including or excluding the MYOF/SRSF10 candidate exon was obtained by fluorescent competitive RT-PCR (see Supplementary Methods for details).

**Figure 4:**
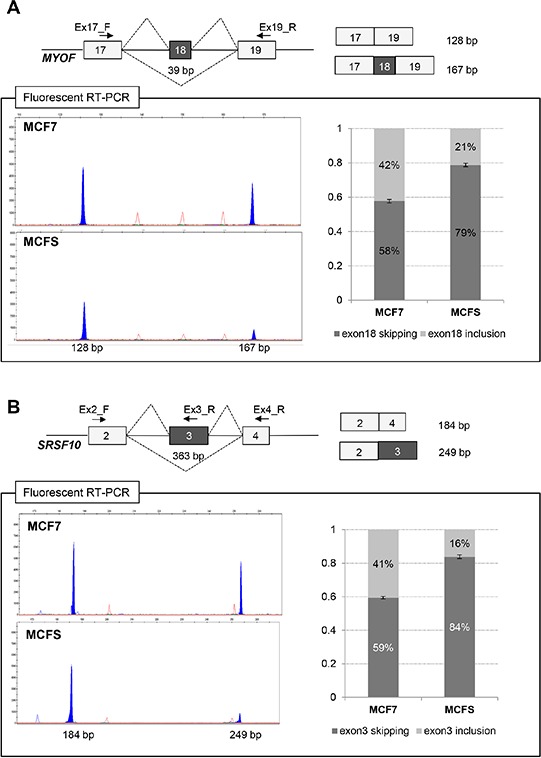
Validation of differentially expressed alternative splicing at the *MYOF* (A) and *SRSF10* (B) loci by fluorescent RT-PCR **A.** Schematic representation of the *MYOF* gene in the region comprised between exons 17 and 19 (top left) and of the two products obtained by fluorescent competitive RT-PCR (top right). Exons are shown by gray boxes, whereas introns are represented by lines (not to scale). Alternative splicing events are shown by broken lines. Primers used for RT-PCRs are indicated by arrows. The length of the fragments is also indicated. GeneMapper windows displaying fluorescence peaks (shaded in gray) corresponding to the different transcripts amplified by fluorescent RT-PCR on RNA from MCF7 cells and MCFS (bottom). The X-axis represents data points and the Y-axis represents fluorescence units. On the right, histograms representing the relative amount of transcripts including or skipping exon 18, as assessed by calculating the ratio of the corresponding fluorescence peak areas (setting the sum of all peaks as 100%). Bars represent mean ± SD of 3 independent experiments. **B.** Schematic representation of the *SRSF10* gene in the region comprised between exons 3 and 4 (top left) and of the two products obtained by fluorescent competitive RT-PCR (top right). GeneMapper windows displaying fluorescence peaks (shaded in gray) corresponding to the different transcripts amplified on RNA from MCF7 cells and MCFS cells (bottom). On the right, histograms representing the relative amount of transcripts including or skipping exon 3, calculated as described above. Bars represent mean ± SD of 3 independent experiments.

We confirmed that inclusion of the in-frame 39-base pair (bp) exon 18 was reduced in MCFS compared to MCF7 cells (from 42% to 21%), thus generating an imbalance in the levels of the two alternatively spliced isoforms (NM_013451 - myoferlin isoform a, including exon 18; NM_133337 - myoferlin isoform b, excluding exon 18) (Figure 4A). Structurally, myoferlin contains 6 tandem C2 domains, designated as (C2A-C2F), a central DysF domain, and a single C-terminal transmembrane region. Each C2 domain folds into an 8-stranded beta-sandwich and usually contains a calcium-binding region. The two myoferlin isoforms, a and b, differ for 13 amino acids located within a loop region of the C2C domain. Interestingly, the C2C domain is the most divergent domain between the different members of the ferlin family of proteins, and it was suggested that the presence of the domain has influenced the functional adaptation of its neighboring domains [35].

We also validated a significant reduction of SRSF10 expression in MCFS and a concomitant imbalance in the inclusion of the in-frame 363-bp exon 3, which decreased from 41% to 16% (Figure 4B). Splicing factor SRSF10 is an atypical member of the serine/arginine-rich family of proteins that can function as a sequence-dependent splicing activator [36]. These regulatory proteins can modulate both exon activation and repression *in vivo*, which is likely dependent on their binding location within the pre-mRNA. Using this strategy, the RNA-binding protein regulates splicing in the cell [37, 38].

**Figure 5:**
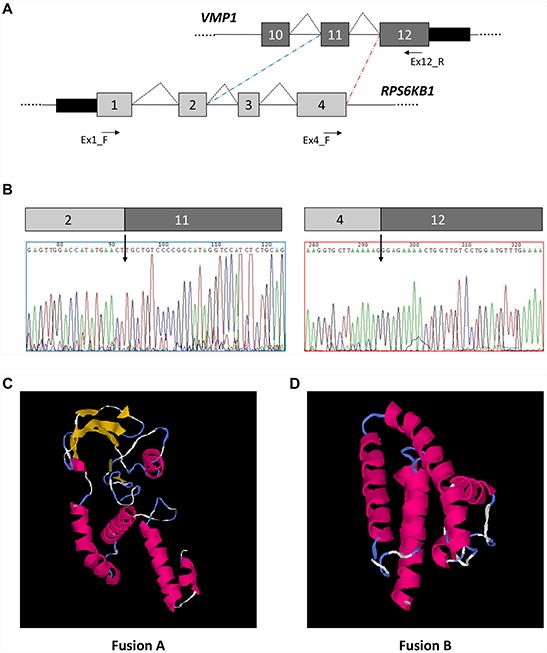
Identification of *RPS6KB1-VMP1* gene fusion **A.** Schematic representation of the *VMP1* and *RPS6KB1* genes in the region comprised between exons 1 and 4 (*RPS6KB1*) or exons 11 and 12 (*VMP1*). Coding exons are shown by gray boxes, whereas introns are represented by lines (not to scale). Splicing events are shown by broken lines, whereas fusions are indicated by dashed lines. Primers used for RT-PCRs are indicated by arrows. **B.** Electropherograms showing the nucleotide sequences around the identified fusion junctions. **C.** Molecular model of fusion A (*RPS6KB1* exons 1 to 4 fused to *VMP1* exon 12). **D.** Molecular model of fusion B (*RPS6KB1* exons 1 and 2 fused to *VMP1* exon 11)

In parallel, to better characterize the predicted RPS6KB1-VMP1 fusion events, we performed RT-PCR using a reverse primer in VMP1 exon 12 and a forward primer located either in RPS6KB1 exon 1 or in exon 4. Using the forward primer in RPS6KB1 exon 4, we obtained a unique amplification product of about 150 bp, whereas using the forward primer in exon 1 we amplified two different products of about 430 and 340 bp, respectively. The direct sequencing of individual RT-PCR products allowed us to identify two different fusion transcripts, one including RPS6KB1 exons 1 to 4 and VMP1 exon 12 (fusion A), and the other connecting RPS6KB1 exons 1 and 2 to VMP1 exon 11 (fusion B)(Figure 5A–5B). The molecular model structure predictions derived from the two open reading frames reconstructed from the fusions, performed with the I-TASSER software [39], suggest that the first fusion could maintain an activity of interaction with ATP and an A-kinase activity, whereas the second fusion could result in a more homogenous multiple alpha-helix structure which maintains only a generic protein amino acid binding function, as identified from GO term association (Figure 5C–5D).

RPS6KB1 is a serine/threonine-protein kinase, an important regulator of cell size control, protein translation, and cell proliferation [40] and it is one of the best characterized downstream targets of mTOR.

Vacuole membrane protein 1 (VMP1) is a plasma membrane protein and an essential component of initial cell–cell contacts and tight junction formation. It has been described as a cancer-relevant cell cycle modulator, but the function of the protein and its mode of action in tumor progression are still unknown. Its high expression is correlated with noninvasive breast cancer cell lines [41].

Our RT-PCR data suggest that the fusion transcript (RPS6KB1-VMP1) is expressed more in MCF7 cells than in MCFS, as suggested by the AltAnalyze analysis.

Interestingly, Inaki et al. [42] found the expression of transcript fusion between RPS6KB1 and VMP1 in 70 breast primary tumors from Singaporean patients and they found that such fusion was expressed in 30% of breast cancers [42]. They found additional fusions points, including the same fusions points we found between RPS6KB1 exon 2 and VMP1 exon 11 and RPS6KB1 exon 4 and VMP1 exon 12. Such gene fusion is caused by tandem duplication of 17q23. In fact, in breast cancer this chromosomal region is frequently amplified.

### Genes overexpressed in MCFS are associated with resistance to endocrine therapy in patients

**Figure 6:**
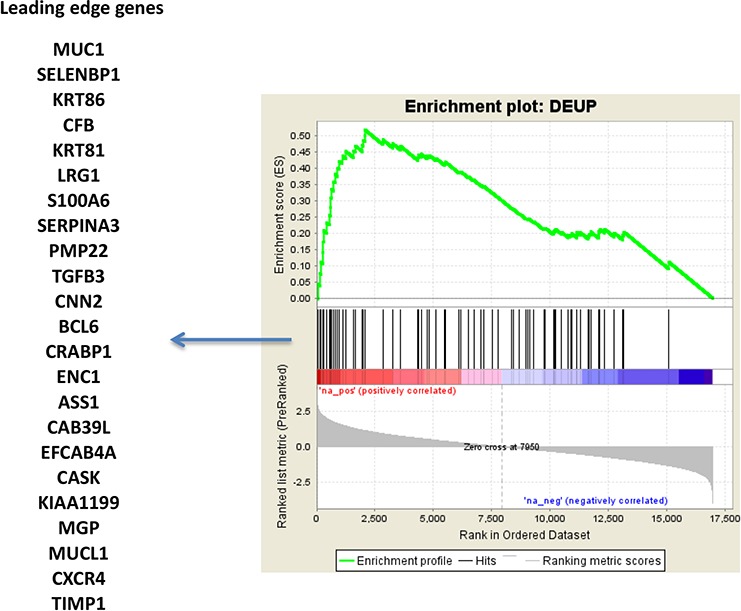
TPC signature in the NEWEST cohort Gene set enrichment analysis of 77 genes concordantly overexpressed in both microarray and RNA-seq data, in MCFS with respect to MCF7 cells. The gene set was evaluated in the NEWEST dataset (GSE48905), contrasting resistant versus responder cases after treatment with fulvestrant.

One of the hypotheses suggested by the interpretation of differentially modulated coding genes was a reduced sensitivity to endocrine treatment of MCFS. To evaluate whether the gene expression modulation observed in this model could also be traced in clinical samples of breast cancer, a consensus list of 77 genes (Supplementary Table S5) overexpressed in MCFS in both array and RNA-seq data was derived. The 77-gene list was evaluated in a public dataset (GSE48905) of gene expression profiles of breast cancers from patients enrolled in the NEWEST (Neoadjuvant Endocrine Therapy for Women with Estrogen-Sensitive Tumours) trial comparing the clinical and biological activity of fulvestrant, 500 mg vs 250 mg, in the neoadjuvant setting [43]. Interestingly, our signature of genes overexpressed in MCFS was enriched in resistant tumors (stable or progressive disease) compared to responders (Figure 6). Such data, combined with our *in vitro* findings, are clinically relevant since fulvestrant has been approved as a second-line therapy for patients experiencing recurrence after a tamoxifen regimen, as it lacks cross-resistance with other antiestrogens, has no agonistic activity and accelerates degradation of ERα. Our results question the utility of fulvestrant as second-line endocrine therapy and suggest that treatment with this pure antiestrogen might fail due to the outgrowth of TPC cell subpopulations.

### Distinguishing between cell-line dependent and culture-condition dependent differences

Finally we asked whether the particular condition for MCFS isolation could have influenced the gene expression pattern. In fact, most studies trying to tackle transcriptome variations in TPCs compared to parental cells have employed enrichments based on the expression of selected markers [24, 27, 31], whereas we chose another largely used approach that is to enrich for TPC by modifying the growth conditions. As a consequence MCFS cells might be regarded as 3D cultures since they form mammospheres whereas MCF7 cells as 2D cultures growing in adhesion conditions. To evaluate the extent of this possible bias, we took advantage from Kenny and colleagues study correlating morphologies and gene expression profiles of 24 breast cancer cell lines grown in 2D versus 3D conditions [44]. We extrapolated from the study 3D versus 2D gene expression profiles for MCF7 cells only and 545 unique genes which showed a fold change larger than 2 (271 up-regulated and 274 down-regulated, Supplementary Table S7). Of our 77-gene signature (all up-regulated genes), 15 genes were up-regulated also in Kenny's study (19% overlap) and 3 were down-regulated, suggesting that 3D culture might have affected gene expression, but that it was not the main driver for the differences identified in our study. Moreover, none of the key genes investigated in this study was differentially expressed in Kenny's study. When considering all the 25 cell lines included in their study, Kenny and colleagues identified 41 genes differentially expressed in 2D versus 3D cells, and a statistically significant overexpression of 1 out of 8 identified Gene Ontology classes, i.e ‘signal transducer activity’. These results suggest that in 3D cultures the main differences relate to regulation of signal transduction, whereas we identified many more biological functions enriched in MCFS compared to MCF7 cells. In our analysis ‘Signal transduction’ is not even among the main enriched biological functions and only appears to be limited to ‘growth factor response’ (Table 1). This is again an indirect evidence for the fact that the distinct MCFS gene expression pattern is cell line- rather than culture condition-dependent. Unfortunately, no external data are available for a similar evaluation of lncRNAs and alternative splicing findings, although a similar pattern is expected.

We also would like to emphasize that the lack of growth of MCFS cells upon stimulation with 10–8 M E2 is not a simple result of culture conditions since in the presence of a serum –free medium supplement (2% XerumFree™ XF205, TNC Bio BV, Eindhoven, NL), estradiol was still stimulatory for MCF7 (Supplementary Figure S2). This is a further direct evidence for the fact that it is not the culture condition that dictates the biological function, but that our TPC enrichments method does in fact select a different cell subpopulation.

In an interesting paper on primary glioblastoma [45], single-cell RNA-seq was used to collect clues on intratumoral heterogeneity in clinical tumors. In parallel, subpopulations of stem like cells (GSCs) were modeled *in vitro* as spherogenic cultures that initiate tumors in mice and their transcriptomes were compared to more differentiated cells expanded as adherent monolayers in serum (i.e. exactly our approach). The derived stemness consensus signature when applied to the single cell transcriptional profiles, revealed a gradient of stemness among the single cells, suggesting that *in vitro* models do in fact give important clues, although limiting to the phenotypic extremes of cellular states. We have not yet proven such a concept in breast, but results on bulk tumors go in this direction.

## MATERIALS AND METHODS

### Cell cultures

The MCF7 breast cancer cell line was purchased from the American Type Culture Collection (ATCC; Manassas, VA, USA) and cultured in DMEM/F-12 (Lonza, Slough, UK) medium supplemented with 10% fetal bovine serum (Lonza). MCFS were derived from the MCF7 cell line [21] and propagated as floating mammospheres in mammary epithelial cell growth medium, an appropriate growth medium composed of MEBM (Lonza) supplemented with B27 without vitamin A (Life Technologies, Foster City, CA, USA), heparin 0.6 U/ml (Eparina Vister 5,000 U/ml), human recombinant epidermal growth factor, 20 ng/ml (Peprotech, NJ, USA) and human recombinant basic fibroblast growth factor, 10 ng/ml (Peprotech). Both cell lines were cultured at 37°C in humidified 5% co2 atmosphere. Cell vitality was assessed by the trypan blue exclusion assay (at least 95%) before starting any experiment. Authentication of cell lines by short tandem repeat DNA profiling analysis was performed at the Niguarda Ca' Granda Hospital in Milan.

### Total RNA extraction

Cell pellets (∼106 cells) were immediately put on ice and homogenized in 1 ml of TRIzol^®^ Reagent (Life Technologies); lysates were stored at −80°C for not more than 2 weeks. Total RNA was extracted following the manufacturer's instructions. Contaminating DNA was digested using recombinant DNase I (Life Technologies), and RNA samples were purified using the RNeasy MinElute Cleanup Kit (Qiagen, Germantown, Maryland, USA). Total RNA was quantified by a NanoDrop spectrophotometer and assessed for quality by the Agilent RNA 6000 Nano kit (Agilent Technologies, Santa Clara, CA, USA) using the Agilent 2100 Bioanalyzer (Agilent Technologies).

For RNA-seq experiments, total RNA from 5000 MCF7 cells and MCFS were extracted following Agencourt^®^ RNAdvance^®^ Cell v2 (Beckman Coulter, Danvers, MA, USA) protocol using Agencourt's patented SPRI^®^ paramagnetic bead available in the kit. After Proteinase K cell lysis and protein digestions, total RNA was separated from contaminants exploiting its binding to the magnetic particles. Manufacturer instructions were followed with the exception of the DNase treatment step, which was carried on with the Ambion^®^ TURBO DNA-free™ (Life Technologies). Fifty μl of a solution containing 1 μl of TURBO DNase (2 Units/μl), 5 μl of 10X TURBO DNase Buffer, and 44 μl of nuclease-free water was prepared, added directly to the bead-linked RNA and incubated at 37°C for 30 min. The addition of aqueous DNase released DNA and RNA from the beads; while DNA was digested, RNA was re-bound to the beads by adding 250 μl of Agencourt Wash Buffer. Thereafter, as in the original Agencourt protocol, RNA was eluted in 40 μl of nuclease-free water and quantified using Qubit™ RNA Assay Kits (Life Technologies); its quality was checked using the Agilent^®^ RNA 6000 Pico kit (Agilent Technologies, Santa Clara, CA, USA).

### Microarray hybridization and analysis

Array experiments with the Illumina platform were run by the Functional Genomics Core Facility at the Fondazione IRCCS Istituto Nazionale Tumori, as previously described [46]. Briefly, RNA derived from frozen samples was processed for microarray hybridization using the Illumina BeadChips HumanRef-8 V3 kit. Total RNA (800 ng) was reverse transcribed, labeled with biotin and amplified overnight using the Illumina RNA TotalPrep Amplification kit (Life Technologies) according to the manufacturer's protocol. The biotinylated cRNA sample (1 μg) was mixed with the Hyb E1 hybridization buffer containing 37.5% (w/w) formamide and then hybridized at 58°C overnight to the Illumina BeadChips HumanRef-8 V3 (Illumina, San Diego, CA, USA). Array chips were washed with the manufacturer's E1BC solution, stained with 1 μg/ml Cy3-streptavidine (Amersham Biosciences, Buckinghamshire, UK), and eventually scanned with the Illumina BeadArray Reader.

Three replicates were profiled for both cell types. Microarray raw data where generated using the Illumina BeadStudio 3.8 software and processed using the *lumi* package [47] of Bioconductor. After quality control, the Robust Spline Normalization was applied, and probes with a detection *P* < 0.01 in at least 2 of 6 samples were selected (11406 probes). When multiple probes per gene were present, only the probe with the highest detection rate or highest interquartile range was retained (9,283 probes). Raw and processed data were deposited at the Gene Expression Omnibus data repository (GSE58383).

### SOLiD library construction and sequencing

Total RNA (90 ng) was amplified, prior to transcriptome sequencing, using Ribo-SPIA^®^ technology developed by NuGEN following Ovation RNA-seq (2009, NuGEN^®^ Technologies, San Carlos, CA, USA). Briefly, first-strand cDNA was prepared from total RNA using unique primers that hybridize either to the 5′ portion of the poly(A) sequence or randomly across the transcript. The resulting mRNA within the cDNA/mRNA complex was fragmented, and DNA was amplified using a linear isothermal DNA process developed by NuGEN named SPIA^®^. The post-SPIA modification process completed the amplification step, producing targets appropriate for SOLiD library preparation. The amplified cDNA was purified prior to subsequent processing for library construction using RNA clean purification magnetic beads (Beckman Coulter Genomics), as suggested in the protocol. Double-stranded DNA was quantified with Qubit™ DNA BR Assay Kits (Life Technologies), and its size distribution was checked using the Agilent^®^ RNA Nano kit (Agilent) following the manufacturer's instructions.

DNA libraries, one for each sample, were constructed following the SOLiD™ 3 Plus System Library Preparation Guide (Life Technologies) manufacturer's instructions. One μg of cDNA was diluted in 100 μl in 1X low TE buffer and transferred in a Covaris™ microTUBE. DNA was sheared using the following Covaris S2 System conditions (Covaris, Woburn, MA, USA): 10 cycles 60 s each, 10% duty cycle, 5 intensity and 100 cycles/burst. Sheared samples were first end-repaired, columns purified and ligated to specific Solid adaptors containing P1 and P2 sequences. DNA was size-selected using a SOLiD™ Library Size Selection gel run in the E-Gel^®^ iBase™ system: the 150–200 bp region was collected.

The nick-translated adaptor-ligated DNA was amplified using Library PCR Primer P1 and P2 with 6 cycles, and after SOLiD™ Library Column purification, the yield and size distribution of the libraries were checked using the Agilent^®^ DNA 1000 Kit (Agilent Technologies). Four PCR emulsions, two for each library, were manually performed following the Applied Biosystems SOLiD™ 3 Plus System Templated Bead Preparation Guide, according to the manufacturer's instructions (Life Technologies).

The library and emulsion qualities were checked in a workflow analysis run following the SOLiD™ 3 Plus System Instrument Operation Guide (Life Technologies).

Sequencing was done using standard fragment settings on the SOLiD™ Systems V3 plus according to SOLiD™ 3 Plus System Instrument Operation Guide protocol (Life Technologies). At least 150 M of tags for each library, 50 bp long, distributed in 2 quad each, were sequenced.

### RNA-seq data processing

Sequence reads in SOLiD Color Space format were mapped to the UCSC repeat-masked hg19 reference genome and analyzed for transcript representation in RPKM and splice site-fusion representation with the Whole Transcriptome Analysis module of the Lifetech Lifescope 2.5.1 analysis software and ad hoc created perl scripts. The resulting genome alignment files in .bam format were used to originate the fastq files corresponding to the aligned sequence reads and for further analysis with the TopHat/Cufflink/Cuffdiff whole transcriptome analysis pipeline.

### Gene set enrichment analysis

Enrichment analysis in mRNA expression data was performed using GSEA (v. 2.0) [48]. The C2 collection (v. 3.1) containing 4,850 gene sets collected from various sources such as online pathway databases, publications in PubMed, and knowledge of domain experts, was tested for enrichment on both microarray and RNA-seq data. Microarray data were ranked according to default signal to noise GSEA metrics or according to fold change. RNA-seq data were ranked according to fold change or, for the Seqsolve processed data, according with the Cuffdiff statistics. Only gene sets for which a number of genes > 15 and < 300 was found in the data were tested. Gene sets with a false discovery rate of < 1% were considered significantly enriched.

### Alternative splicing analysis

To identify splicing events in MCFS compared with the parental MCF7 cells, AltAnalyze software [34] was used, starting from the exon-level quantification and exon junction tables generated by the Lifescope pipeline. A reciprocal junction analysis, which identifies pairs of exon-exon junctions differentially expressed in opposite directions in the two cell lines, was performed. Such a method is reported to be very accurate at detecting true alternative splicing events. Statistical significance was assessed using the ASPIRE score, and events with ASPIRE > 0.2 or < 0.2 were considered significant. A combination of filtering criteria was applied to the more than 2000 significant events in order to select a more limited number of biologically relevant elements. The filtering procedure is summarized in Supplementary Figure S1.

First of all, we selected events occurring in genes expressed at relatively high levels, i.e., RPKM > 5 in MCFS and MCF7 cells. Then, we distinguished between events with positive or negative ASPIRE values. In exon junctions, positive ASPIRE values correspond to a down-regulation of junction 1 and up-regulation of junction 2, and vice versa for ASPIRE negative values. The subsequent criterion was to have a value higher than 15 in the sample were the junction was up-regulated. For events in unchanging genes, we also selected events where fold change value was higher than 3.

See Supplementary Methods and Supplementary Table S6 for description of experimental validations.

## CONCLUSIONS

We performed a detailed study of TPC transcriptome using microarrays and RNA-seq. Informative RNA-seq data were derived starting from small input RNA, making the approach applicable to various scenarios where a limited amount of material is available. Some of our findings were already reported as being crucial in TPC, although using different approaches to isolate such a tumor subpopulation, therefore supporting the validity of our model. Of note was the up-regulation of the NF-κB pathway, IL-8 and other inflammatory cytokines. Our TPC also showed less responsiveness to endocrine treatment, and, interestingly, genes over-expressed in MCFS cells were found to be up-regulated also in clinical tumors resistant to fulvestrant treatment, suggesting that they might represent a new putative predictive marker of hormone-treatment resistance. Finally, RNA-seq analysis suggested an involvement of several ncRNAs and differential splicing events in maintenance of the TPC phenotype.

We are aware that it is not yet clear if the putative stem cell components derived from established cell lines is a valid model for TPC and that experimental conditions might be very critical, however, i) the confirmation of some earlier literature data [27, 31], ii) the *in vitro* validation of the hypothesis generated by the data and iii) the successful identification of a gene signature predicting response to fulvestrant obtained in this study, give additional strength to other still to validate findings and to the model itself.

## SUPPLEMENTATRY METHODS FIGURES AND TABLES











## References

[1] O'Connor ML, Xiang D, Shigdar S, Macdonald J, Li Y, Wang T, Pu C, Wang Z, Qiao L, Duan W (2014). Cancer stem cells: A contentious hypothesis now moving forward. Cancer Lett.

[2] Ablett MP, Singh JK, Clarke RB (2012). Stem cells in breast tumours: are they ready for the clinic?. Eur J Cancer.

[3] Wicha MS, Liu S, Dontu G. (2006). Cancer stem cells: an old idea--a paradigm shift. Cancer Res.

[4] Fillmore CM, Kuperwasser C (2008). Human breast cancer cell lines contain stem-like cells that self-renew, give rise to phenotypically diverse progeny and survive chemotherapy. Breast Cancer Res.

[5] Angeloni V, Tiberio P, Appierto V, Daidone MG (2014). Implications of stemness-related signaling pathways in breast cancer response to therapy. Semin Cancer Biol.

[6] Al-Hajj M, Wicha MS, Benito-Hernandez A, Morrison SJ, Clarke MF (2003). Prospective identification of tumorigenic breast cancer cells. Proc Natl Acad Sci USA.

[7] Dontu G, Al-Hajj M, Abdallah WM, Clarke MF, Wicha MS (2003). Stem cells in normal breast development and breast cancer. Cell Prolif.

[8] Dontu G, Jackson KW, McNicholas E, Kawamura MJ, Abdallah WM, Wicha MS (2004). Role of Notch signaling in cell-fate determination of human mammary stem/progenitor cells. Breast Cancer Res.

[9] Androutsellis-Theotokis A, Leker RR, Soldner F, Hoeppner DJ, Ravin R, Poser SW, Rueger MA, Bae SK, Kittappa R, McKay RD (2006). Notch signalling regulates stem cell numbers in vitro and in vivo. Nature.

[10] Liu S, Dontu G, Mantle ID, Patel S, Ahn NS, Jackson KW, Suri P, Wicha MS (2006). Hedgehog signaling and Bmi-1 regulate self-renewal of normal and malignant human mammary stem cells. Cancer Res.

[11] Zhou J, Zhang H, Gu P, Bai J, Margolick JB, Zhang Y (2008). NF-kappaB pathway inhibitors preferentially inhibit breast cancer stem-like cells. Breast Cancer Res Treat.

[12] Murohashi M, Hinohara K, Kuroda M, Isagawa T, Tsuji S, Kobayashi S, Umezawa K, Tojo A, Aburatani H, Gotoh N (2010). Gene set enrichment analysis provides insight into novel signalling pathways in breast cancer stem cells. BrJ Cancer.

[13] Charafe-Jauffret E, Ginestier C, Iovino F, Wicinski J, Cervera N, Finetti P, Hur MH, Diebel ME, Monville F, Dutcher J, Brown M, Viens P, Xerri L (2009). Breast cancer cell lines contain functional cancer stem cells with metastatic capacity and a distinct molecular signature. Cancer Res.

[14] Ginestier C, Liu S, Diebel ME, Korkaya H, Luo M, Brown M, Wicinski J, Cabaud O, Charafe-Jauffret E, Birnbaum D, Guan JL, Dontu G, Wicha MS (2010). CXCR1 blockade selectively targets human breast cancer stem cells in vitro and in xenografts. J Clin Invest.

[15] Borgna S, Armellin M, di GA, Maestro R, Santarosa M (2012). Mesenchymal traits are selected along with stem features in breast cancer cells grown as mammospheres. Cell Cycle.

[16] Mallini P, Lennard T, Kirby J, Meeson A. (2014). Epithelial-to-mesenchymal transition: what is the impact on breast cancer stem cells and drug resistance. Cancer Treat Rev.

[17] Zhang H, Chen Z, Wang X, Huang Z, He Z, Chen Y (2013). Long non-coding RNA: a new player in cancer. J Hematol Oncol.

[18] Katsushima K, Kondo Y (2014). Non-coding RNAs as epigenetic regulator of glioma stem-like cell differentiation. Front Genet.

[19] Salomonis N, Schlieve CR, Pereira L, Wahlquist C, Colas A, Zambon AC, Vranizan K, Spindler MJ, Pico AR, Cline MS, Clark TA, Williams A, Blume JE (2010). Alternative splicing regulates mouse embryonic stem cell pluripotency and differentiation. Proc Natl Acad Sci USA.

[20] Lu Y, Loh YH, Li H, Cesana M, Ficarro SB, Parikh JR, Salomonis N, Toh CX, Andreadis ST, Luckey CJ, Collins JJ, Daley GQ, Marto JA (2014). Alternative Splicing of MBD2 Supports Self-Renewal in Human Pluripotent Stem Cells. Cell Stem Cell.

[21] Ladomery M (2013). Aberrant Alternative Splicing Is Another Hallmark of Cancer. Int J Cell Biol.

[22] Wang Z, Gerstein M, Snyder M (2009). RNA-Seq: a revolutionary tool for transcriptomics. Nat Rev Genet.

[23] Ponti D, Costa A, Zaffaroni N, Pratesi G, Petrangolini G, Coradini D, Pilotti S, Pierotti MA, Daidone MG (2005). Isolation and in vitro propagation of tumorigenic breast cancer cells with stem/progenitor cell properties. Cancer Res.

[24] Hardt O, Wild S, Oerlecke I, Hofmann K, Luo S, Wiencek Y, Kantelhardt E, Vess C, Smith GP, Schroth GP, Bosio A, Dittmer J (2012). Highly sensitive profiling of CD44+/CD24- breast cancer stem cells by combining global mRNA amplification and next generation sequencing: evidence for a hyperactive PI3K pathway. Cancer Lett.

[25] Frasor J, Stossi F, Danes JM, Komm B, Lyttle CR, Katzenellenbogen BS (2004). Selective estrogen receptor modulators: discrimination of agonistic versus antagonistic activities by gene expression profiling in breast cancer cells. Cancer Res.

[26] Frasor J, Danes JM, Komm B, Chang KCN, Lyttle CR, Katzenellenbogen BS (2003). Profiling of estrogen up- and down-regulated gene expression in human breast cancer cells: insights into gene networks and pathways underlying estrogenic control of proliferation and cell phenotype. Endocrinology.

[27] Shipitsin M, Campbell LL, Argani P, Weremowicz S, Bloushtain-Qimron N, Yao J, Nikolskaya T, Serebryiskaya T, Beroukhim R, Hu M, Halushka MK, Sukumar S, Parker LM (2007). Molecular definition of breast tumor heterogeneity. Cancer Cell.

[28] Li X, Lewis MT, Huang J, Gutierrez C, Osborne CK, Wu M-F, Hilsenbeck SG, Pavlick A, Zhang X, Chamness GC, Wong H, Rosen J, Chang JC (2008). Intrinsic resistance of tumorigenic breast cancer cells to chemotherapy. J Natl Cancer Inst.

[29] Woodward WA, Chen MS, Behbod F, Alfaro MP, Buchholz TA, Rosen JM (2007). WNT/beta-catenin mediates radiation resistance of mouse mammary progenitor cells. Proc Natl Acad Sci U S A.

[30] Dixon JM (2014). Endocrine Resistance in Breast Cancer. New J Sci.

[31] Harrison H, Simões BM, Rogerson L, Howell SJ, Landberg G, Clarke RB (2013). Oestrogen increases the activity of oestrogen receptor negative breast cancer stem cells through paracrine EGFR and Notch signalling. Breast Cancer Res.

[32] Singh JK, Simões BM, Howell SJ, Farnie G, Clarke RB (2013). Recent advances reveal IL-8 signaling as a potential key to targeting breast cancer stem cells. Breast Cancer Res.

[33] Guffanti A, Iacono M, Pelucchi P, Kim N, Soldà G, Croft LJ, Taft RJ, Rizzi E, Askarian-Amiri M, Bonnal RJ, Callari M, Mignone F, Pesole G (2009). A transcriptional sketch of a primary human breast cancer by 454 deep sequencing. BMC Genomics.

[34] Emig D, Salomonis N, Baumbach J, Lengauer T, Conklin BR, Albrecht M (2010). AltAnalyze and DomainGraph: analyzing and visualizing exon expression data. Nucleic Acids Res.

[35] Jimenez JL, Bashir R (2007). In silico functional and structural characterisation of ferlin proteins by mapping disease-causing mutations and evolutionary information onto three-dimensional models of their C2 domains. J NeurolSci.

[36] Zhou X, Wu W, Li H, Cheng Y, Wei N, Zong J, Feng X, Xie Z, Chen D, Manley JL, Wang H, Feng Y (2014). Transcriptome analysis of alternative splicing events regulated by SRSF10 reveals position-dependent splicing modulation. Nucleic Acids Res.

[37] Feng Y, Chen M, Manley JL (2008). Phosphorylation switches the general splicing repressor SRp38 to a sequence-specific activator. Nature Struc Mol Biol.

[38] Llorian M, Schwartz S, Clark TA, Hollander D, Tan LY, Spellman R, Gordon A, Schweitzer AC, de la Grange P, Ast G, Smith CW (2010). Position-dependent alternative splicing activity revealed by global profiling of alternative splicing events regulated by PTB. Nature Struct Mol Biol.

[39] Roy A, Kucukural A, Zhang Y (2010). I-TASSER: a unified platform for automated protein structure and function prediction. Nature Protoc.

[40] Fingar DC, Blenis J (2004). Target of rapamycin (TOR): an integrator of nutrient and growth factor signals and coordinator of cell growth and cell cycle progression. Oncogene.

[41] Sauermann M, Sahin O, Sultmann H, Hahne F, Blaszkiewicz S, Majety M, Zatloukal K, Fuzesi L, Poustka A, Wiemann S, Arlt D (2008). Reduced expression of vacuole membrane protein 1 affects the invasion capacity of tumor cells. Oncogene.

[42] Inaki K, Hillmer AM, Ukil L, Yao F, Woo XY, Vardy LA, Zawack KF, Lee CW, Ariyaratne PN, Chan YS, Desai K V, Bergh J, Hall P (2011). Transcriptional consequences of genomic structural aberrations in breast cancer. Genome Res.

[43] Knudsen S, Jensen T, Hansen A, Mazin W, Lindemann J, Kuter I, Laing N, Anderson E (2014). Development and validation of a gene expression score that predicts response to fulvestrant in breast cancer patients. PLoS One.

[44] Kenny PA, Lee GY, Myers CA, Neve RM, Semeiks JR, Spellman PT, Lorenz K, Lee EH, Barcellos-Hoff MH, Petersen OW, Gray JW, Bissell MJ (2007). The morphologies of breast cancer cell lines in three-dimensional assays correlate with their profiles of gene expression. Mol Oncol.

[45] Patel AP, Tirosh I, Trombetta JJ, Shalek AK, Gillespie SM, Wakimoto H, Cahill DP, Nahed B V, Curry WT, Martuza RL, Louis DN, Rozenblatt-Rosen O, Suvà ML (2014). Single-cell RNA-seq highlights intratumoral heterogeneity in primary glioblastoma. Science.

[46] Callari M, Musella V, Di Buduo E, Sensi M, Miodini P, Dugo M, Orlandi R, Agresti R, Paolini B, Carcangiu ML, Cappelletti V, Daidone MG (2014). Subtype-dependent prognostic relevance of an interferon-induced pathway metagene in node-negative breast cancer. Mol Oncol.

[47] Du P, Kibbe WA, Lin SM (2008). lumi: a pipeline for processing Illumina microarray. Bioinformatics.

[48] Subramanian A, Tamayo P, Mootha VK, Mukherjee S, Ebert BL, Gillette MA, Paulovich A, Pomeroy SL, Golub TR, Lander ES, Mesirov JP (2005). Gene set enrichment analysis: a knowledge-based approach for interpreting genome-wide expression profiles. Proc Natl Acad Sci USA.

