# Supplementary material for: In-depth characterization of breast cancer tumor-promoting cell transcriptome by RNA sequencing and microarrays
Source: Oncotarget. 2015 Nov 3;7(1):976–94. doi: 10.18632/oncotarget.5810 (PMC4808046; doi:10.18632/oncotarget.5810)
Supplement: Supplementary file 1 [file oncotarget-07-0976-s001.pdf]

SUPPLEMENTATRY METHODS

Hormone-responsiveness experiments

For experiments with 17β-estradiol (Sigma, St. Louis, Missouri, USA) MCF7 cells were seeded at a 10^4 cell/cm^2 density in Phenol Red free DMEM/F 12 medium supplemented with 5% Charcoal stripped FBS (Gibco, Life technologies, Foster City, CA, USA) and MCFS cells were seeded at a 7×10^3 cells/ml density in Phenol Red-free MEGM. For experiments with ICI 182,780 (fulvestrant, ICI Pharmaceuticals, London, UK) MCF7 cells were seeded at a 10^4 cell/cm^2 density in Phenol Red free DMEM/F-12 medium supplemented with 5% FBS (Lonza) and MCFS cells were seeded at a 7×10^3 cells/mL density in Phenol Red-free MEGM. After 24 hours from seeding, cells were exposed to the specified treatment medium containing either10^{-8} M 17β-estradiol or 10^{-8} M fulvestrant for 6 days and counted after 3 and 6 days of treatment.

MCF7 cells were harvested using TrypLE™ Select 1X (Invitrogen, Life technologies, Foster City, CA, USA), whereas MCFS cells were collected by centrifugation and monodisperse cell suspensions were obtained by enzymatic (TrypLE™ Select 1X) and mechanical dissociation of mammospheres. Cells were centrifuged at 1,000 × *g* for 5 minutes at room temperature and resuspended in medium; then an aliquot of cell suspension was diluted with Isoton II Diluent (Beckman Coulter, Brea, CA, USA). All cell counts were performed with the COULTER COUNTER® (Beckman Coulter).

RNA extraction, quantitative real-time PCR

In order to assess ER-related genes expression, MCF7 and MCFS cells were exposed to 10^{-8} M 17β-estradiol for 48 hours, following the protocol described above. At the end of treatment cells were harvested using TrypLE™ Select 1X and collected by centrifugation in ice cold DPBS 1X (Lonza) at 1,000 × *g* for 5 minutes at 4°C. Cell pellets (~10^6 cells) were immediately put on ice, homogenized in 1 mL of TRIzol® Reagent (Invitrogen) and lysates were stored at −80°C for not more than 2 weeks. Total RNA was extracted following the manufacturer instructions. Contaminating DNA was digested using the Ambion® (Austin, TX, USA) recombinant DNase I and RNA samples were purified using the RNeasyMinElute Cleanup Kit (Qiagen, Germantown, Maryland, USA). Total RNA was quantified by Nanodrop and assessed for quality by the Agilent RNA 6000 Nano kit (Agilent Technologies, Santa Clara, CA, USA) using the Agilent 2100 Bioanalyzer (Agilent Technologies).

cDNA synthesis was performed in a GeneAmp® 9700 PCR System using the High Capacity cDNA Reverse Transcription kit (Applied Biosystems, Life technologies, Foster City, CA, USA) starting from 500 ng of total RNA.

Expression of ER-related genes was assessed using the following TaqMan® assays (Applied Biosystems): Hs00231122_m1 (*GATA3*), Hs00174860_m1 (*ESR1*), Hs00172183_m1 (*PGR*), Hs00157201_m1 (*CTSD*), Hs00907239_m1 (*TFF1*), Hs00536409_m1 (*GREB1*) and Hs00765553_m1 (*CCND1*). *RPL13A* (Hs04194366_g1) was used as housekeeping gene. For each gene 10 ng of cDNA were amplified in triplicate in a 7900HT Fast Real-Time PCR System using the TaqMan® Universal PCR Master Mix (Applied Biosystems). Gene expression fold changes with respect to controls were calculated applying the ΔΔCt method.

Protein extraction and Western blotting

For proteomic analysis, ~10^6 cell pellets were prepared as described for RNA extraction, and immediately stored at −80°C for not more than 1 week. Cells were homogenized at 4°C for 30 minutes in about 30 µl of lysis buffer 1X (50 mMTris-HCl pH 7.4.5 mM EDTA, 250 mM NaCl, 0.1% Triton X-100) supplemented with proteases and phosphatases inhibitors (1 mM PMSF, 5 µg/ml pepstatin A, 10 µg/ml leupeptin, 10 µg/ml aprotinin, 10 mMNaF, 5 mM Na_3VO_4, 5 mM Glycerol-2-Phosphate, 1 mM $Na_4P_20_7$). Proteins were extracted by centrifugation at 12,000 × *g* for 10 minutes at 4°C and stored at –80°C for not more than 2 weeks. Protein quantification was performed using the Micro BCA Protein Assay Kit (Pierce Biotechnology, Rockford, IL, USA), following the manufacturer instructions. Absorbance was measured at 562 nm using the Bio-Rad Microplate Reader (Bio-Rad, Hercules, CA, USA) and protein concentration was estimated using a Bovine Serum Albumin standard curve. Twenty-five micrograms of protein extracts from each sample were denatured in 5X Laemmli sample buffer and used for Western blot analysis. Western blots were performed using a Bio-Rad system. Anti-β-actin antibody was purchased from Abcam (Cambridge, UK) and anti-ERα antibody (HC-20 clone) was purchased from Santa Cruz Biotechnology (Dallas, Texas, USA). Blotted proteins were developed with the ECL Plus chemiluminescence reagent (GE Healthcare, Buckinghamshire, UK) following the manufacturer instructions. The densitometric analysis was performed using the ImageJ software.

Cytokines quantification in cell-conditioned medium

MCF7 cells were seeded at a 10^4 cell/cm^2 density in DMEM/F-12 medium supplemented with 5% FBS; after

24 hours the flask was washed with DPBS 1X and cells were cultured for 4 days in fresh MEGM medium. MCFS cells were seeded at a 7 × 10^3 cells/ml density and cultured in their own growth medium for 4 days. Conditioned media were daily collected by centrifugation at 1,000 × *g* for 5 minutes at 4°C and their volume were measured; in parallel, cells were harvested and counted with the COULTER COUNTER® in order to normalize secreted cytokines concentration with respect to cell numbers. Conditioned media filtered using 45 µm filters (Millipore, Billerica, MA, USA) were stored at -80°C in sub-aliquots for no more than 2 weeks.

IL-8 and MCP-1 quantification were performed using the Human CXCL8/IL-8 Quantikine ELISA Kit and the Human CCL2/MCP-1 Quantikine ELISA Kit (R&D systems, Minneapolis, MN, USA) following the manufacturer instructions. Optical density was measured at 450 nm using a Bio-Rad Microplate Reader (Bio-Rad, Hercules, CA, USA). Plate absorbance (550 nm) was subtracted and cytokines concentrations were estimated using a standard curve. Final cytokines concentrations were calculated and normalized with respect to cell numbers.

Real-time RT-PCR for ncRNA

Random primers and the SuperScript-III reverse transcriptase (Invitrogen Life Technologies, Carlsbad, CA, USA) were used to perform first-strand complementary DNA (cDNA) synthesis starting from 50 ng of total RNA, according to the manufacturer's instructions. Specific primer couples to amplify ncRNAs of interest were designed using Primer3Plus [1] and are listed in Supplementary Table 6. Semi-quantitative real-time RT-PCR reactions (12.5 µL) were performed in triplicate using the 2x SYBR Premix Ex Taq II (Takara Bio Inc, Shiga, Japan) on a Light Cycler 480 (Roche, Basel, Switzerland) starting from 1 µl of a 1:2 dilution of the cDNA template. The touchdown protocol for PCR amplification can be provided on request. Expression levels were normalized using the glucuronidase (*GUSB*) housekeeping gene, and analyzed applying the ΔΔCt method. An unpaired, two-tailed *t*-test was performed to test for significant differences between the two cell types.

Fluorescent RT-PCR

Total RNA was reverse-trascribed using the High-Capacity Reverse Transcription Kit (Applied Biosystem, CA, USA).

To quantify splice products, fluorescent competitive RT-PCRs were performed on RNA from MCF7 and MCFS cells. An aliquot (5 ng) of the total cDNA was used as template in a standard RT-PCR amplifications using fluorescein-labeled exonic forward primers. For the analysis of *MYOF* exon 18 (39-bp long) alternative splicing, primers were designed to amplify a region spanning from exon 17 to 19 (MYOF-Ex17F-FAM and MYOF-Ex19R) (Supplementary Table 6). Instead, to quantify the relative amount of transcript including or skipping *SRSF10* exon 3 (363-bp long), a fluorescein-labeled forward primer was designed in exon 2 and coupled in the RT-PCR reactions with emimolar amount of two different reverse primers: one was located within exon 3, thus selectively amplifying exon 3-including transcript, and one located within exon 4, (Supplementary Table 5) which –in competitive amplification conditions - exclusively amplified exon 3-skipping mRNAs.

RT-PCRs were carried out under standard conditions using the HotStart Taq Master Mix kit (QIAGEN) on a Programmable Thermal Controller-100 (GENENCO). The RT-PCR products were resolved by capillary electrophoresis on an ABI-3130XL Genetic Analyzer (Applied Biosystems). The relative amount of each splicing product was determined by measuring the peak area (evaluated by the GeneMapper v4.0 software) and dividing it by the sum of all peak areas detected in the same PCR reaction. Because the two PCR products are amplified by the same primers, and the two amplicons have similar amplification efficiencies, the ratio of amplified products reflects the relative abundance of the templates before PCR.

Experimental validation of gene fusions

For the analysis of *VMP1-RPS6KB1* fusions, two different forward primers were designed within *RPS6KB1* exon 1 and 4, and a reverse primer within *VMP1* exon 12 (Supplementary Table 6). RT-PCRs were carried out under standard conditions using the HotStart Taq Master Mix kit (QIAGEN) on a Programmable Thermal Controller-100 (GENENCO). Amplified product were purified using the Wizard SV Gel and PCR Clean-Up System (Promega), following the manufacturer's protocol, and subjected to direct DNA sequencing on an ABI-3130XL Genetic Analyzer (Life Technologies, Foster City, CA, USA), as previously described [2].

SUPPLEMENTATRY FIGURES AND TABLES

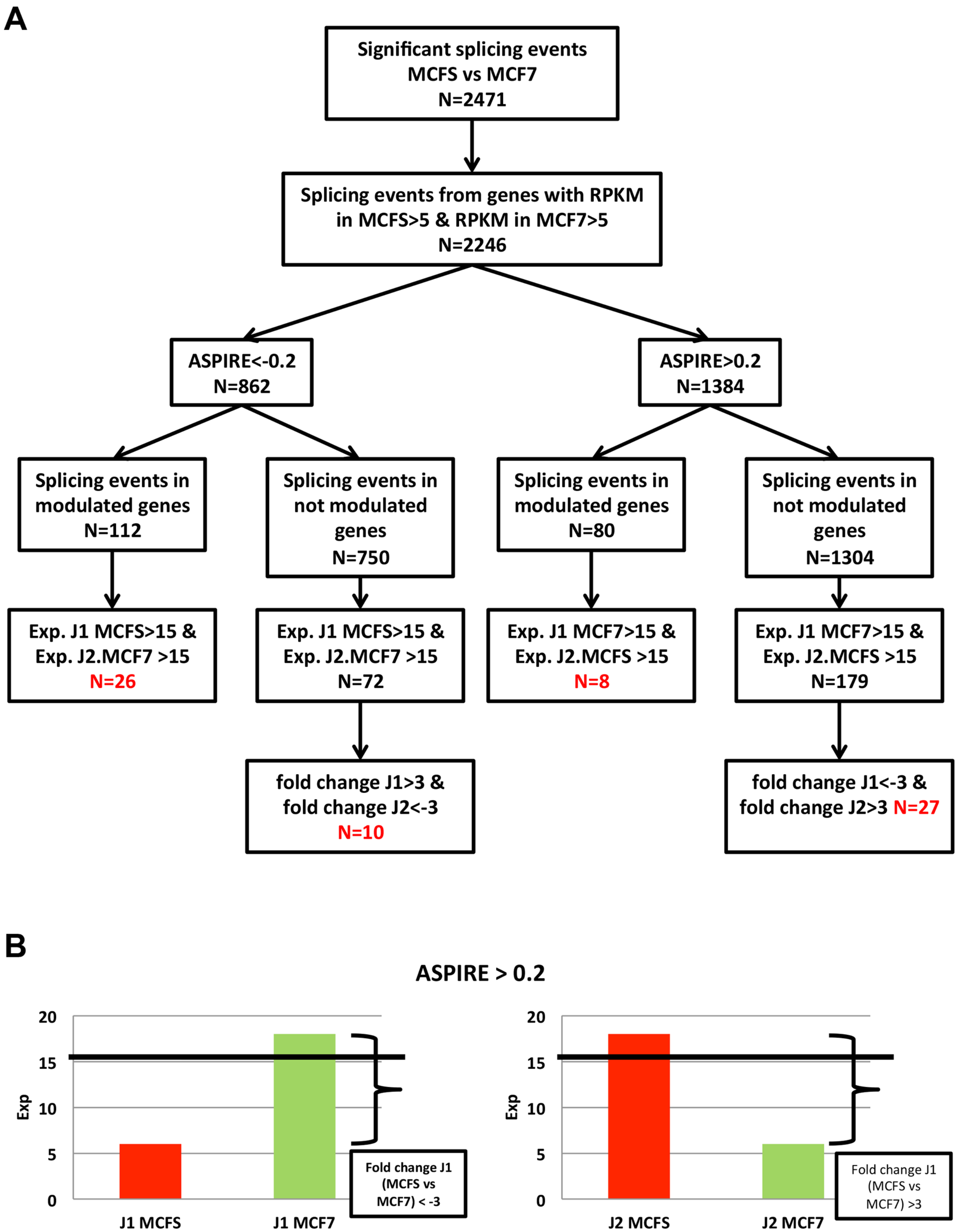

Supplementary Figure S1: Filtering Criteria applied to AltAnalyze output.

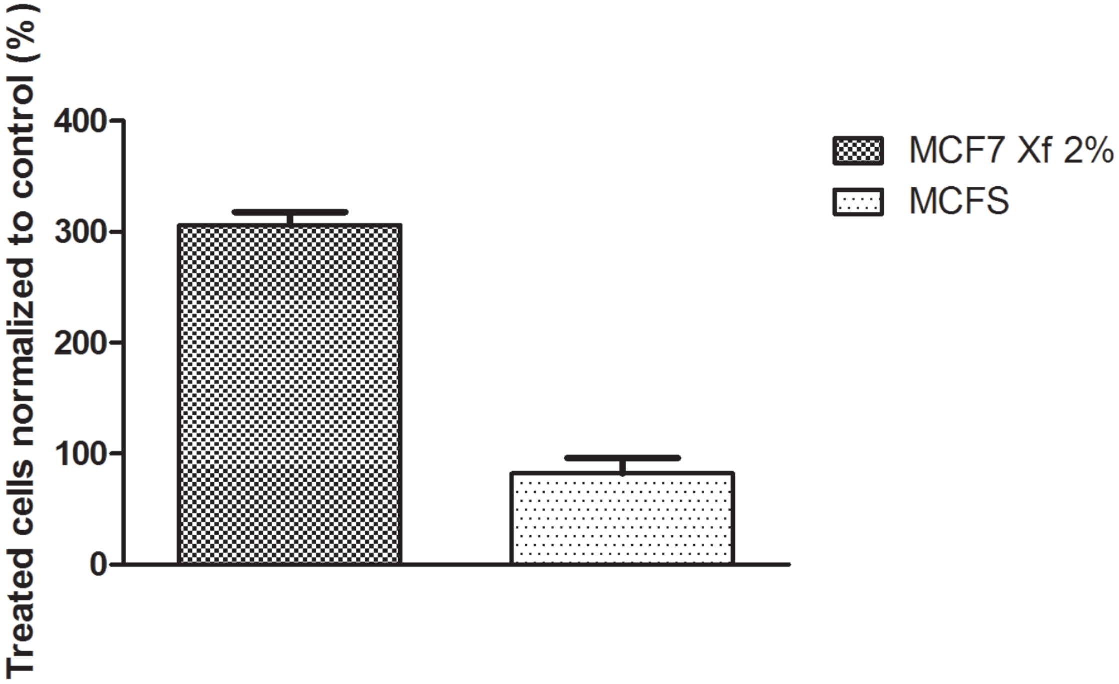

Supplementary Figure S2: Different response to estradiol in MCF7 and MCFS cells is not a consequence of culture conditions. Cells were treated with10^{-8} M 17β-estradiol for 6 days in serum-free conditions: DMEM/F-12 plus 2% serum-free hormone-free medium supplement (XerumFree™ XF20, TNC Bio BV, Eindhoven, NL) for MCF7 and MEGM for MCFS. Response to estradiol was evaluated by direct cell counting (Beckman Coulter Counter) and cell growth response was expressed as percentage the number of treated cells with respect to the number untreated cells. Bar charts represent a mean ± CV% of 3 experimental replicates.

Supplementary Table S1: Number of genes with non-zero counts in RNA-seq data using Lifescope or Seqsolve pipeline

Cell line	Lifescope		Seqsolve		Common
	# Genes	Union	# Genes	Union	
MCFS	16388	18476	14574	17338	16324
MCF7	17591		16324		

Supplementary Table S2: Summary of Gene Sets Enrichment Analysis results

	Genes	Gene sets enriched in MCFS*	Gene sets enriched in MCF7*	Tested**
**Array SNR**	9283	90	92	2979
**Array FC**	9283	79	97	2979
**Lifescope FC**	7131	50	81	2569
**Cufflinks Test**	7120	52	123	2496
**Cufflinks FC**	7120	53	78	2496

*FDR<1%
**Only gene sets with a number of genes in the dataset >15 or <300 were tested

Supplementary Table S3: List of differentially expressed ncRNAs in the comparison between MCFS and MCF7.

Supplementary Table S4: Differentially expressed splice junctions after filtering.

Supplementary Table S5: List of genes up-regulated in both microarray and RNA-seq data (77-gene signature).

Supplementary Table S6: List of primers used for RNA-seq data validation

Gene	Forward primer (5′ → 3′)	Reverse primer (5′ → 3′)	Amplicon(s) size (bp)	Application
**PVT1**	CCGGACGACTCTGACATTTT	TCAAGTCCGTCCAGAGTGCT	109	Real-time RT-PCR
**SNHG3**	ACGGAGTCGGTTTGTCACTC	TGCAGATCGTCTTCTCTCCA	159	Real-time RT-PCR
**MALAT1**	GCTTGAGGAAACCGCAGATA	CGTTAAAAACTTAACGCTAAGCAA	116	Real-time RT-PCR
**RMST**	TCCCATAGAACCACGGAGAC	AGCACAGGGCTCTAACAGGA	153	Real-time RT-PCR
**LINC00673**	CGTGGTGGAATCAGAGGTTT	AACTTCCTGGTGGGAATGTG	104	Real-time RT-PCR
**NR_015353**	TGGCAAGCATTGTAGACCTG	CTTGTTAGGCACAGCGAGGT	130	Real-time RT-PCR
**GUSB**	AAACGATTGCAGGGTTTCAC	CTCTCGTCGGTGACTGTTCA	171	Real-time RT-PCR
**MYOF**	[6FAM]-CTAAAATTGCTGCCTCTGGTG	TGGGAATCCCGTGTACTCTC	167, 128	Fluorescent RT-PCR
**SRSF10**	[6FAM]-GGCGTGAATTTGGTCGTTAT	CTTTGGCTTGTCTACTTGCCTTA &CCCTGGGCAAACTGTATTTC	249, 184	Fluorescent RT-PCR
**RPS6KB1-VMP1 (ex4-ex12)**	ATTTGCCATGAAGGTGCTTA	TCTGAGTTCAACCGCTGCT	154	RT-PCR
**RPS6KB1-VMP1 (ex1-ex12)**	ATAGACCTGGACCAGCCAGA	TCTGAGTTCAACCGCTGCT	427, 340	RT-PCR

Supplementary Table S7: Log2 fold changes comparing 3D versus 2D MCF7 gene expression profiles from Kenny and colleagues study [44]. Log2 fold changes larger than 1 are in red and log2 fold changes less than -1 are in green. Overlap or not with our 77-gene signatures is reported.
